# Aortic rupture from MSSA aortitis in a healthy 21-year-old: a case report

**DOI:** 10.1186/s12879-025-11779-5

**Published:** 2025-12-05

**Authors:** Johannes Kinzinger, Konstanze Horke, Michael Schmoeckel, Dietmar Wassilowsky

**Affiliations:** 1 Department of Anesthesiology, LMU Hospital, Ludwig Maximilian University, Munich, Germany; 2 Department of Cardiac Surgery, LMU Hospital, Ludwig Maximilian University, Munich, Germany

**Keywords:** Aortitis, Staphylococcus aureus bacteremia, Aortic rupture, MSSA

## Abstract

**Background:**

Infectious aortitis is a rare disease affecting predominantly older individuals with pre-existing conditions. We report an unusual case of a healthy, athletic 21-year old male, who developed extensive aortitis with consecutive aortic rupture.

**Case presentation:**

A 21-year old male reported to the emergency department of a local hospital with persisting symptoms of a respiratory tract infection. After admission and initial antibiotic therapy symptoms aggravated and the patient presented with staphylococcus aureus (methicillin-susceptible, MSSA) bacteraemia and a pericardial effusion. After the operative drainage, a routine CT-scan showed a rupture of the aortic arch. The patient was transferred to our hospital and operated. Histopathological analysis of resected aortic tissue showed a phlegmonous inflammation of the aortic wall with the proof of MSSA. The patient recovered completely and was discharged after 22 days.

**Conclusion:**

A case of infectious aortitis in a young individual without any pre-existing condition or pathology of the aorta is extremely uncommon and unlikely. The clinical diagnosis of aortitis is difficult due to its unspecific presentation. Predisposing factors like age, pre-existing conditions, pathologies of the aorta and immunosuppression are described in literature and can help to raise suspicion. This unique case shows infectious aortitis can develop even in the absence of all described risk factors.

**Supplementary Information:**

The online version contains supplementary material available at 10.1186/s12879-025-11779-5.

## Background

Infectious aortitis is the more uncommon subtype amongst inflammatory diseases of the aortic wall, affecting predominantly older individuals with pre-existing aortic pathologies. Due to the often non-specific presentation, diagnosis is difficult and mortality high, especially if untreated [1]. Specific epidemiologic data for infectious aortitis does not exist to our knowledge, considering the rarity this seems not unlikely. Several case reports and case studies over the last decades helped to characterize this entity, but our case, due to its fundamental difference, adds a completely new perspective [2–7]. Here, we present the case of a healthy and athletic 21-year old man, who suffered a contained rupture of the aortic arch due to extensive inflammatory tissue damage caused by a staphylococcus aureus aortitis. We report the perioperative management of this case and discuss possible etiology.

## Case presentation

A 21-year old male patient, with no relevant medical history, presented to the emergency department of the referring hospital (general care) with fatigue, fever, breath- and movement dependent thoracic pain radiating to the left ear and shoulder region. The patient had just returned from a bike tour of several hundred kilometers across the Alps, prior to which he reported having already a cold and sore throat. The patient was admitted to a pulmonology ward and treated empirically for pneumonia with azithromycin. Because of persisting fever over four days under antimicrobial therapy, antibiotic treatment was switched to ampicillin/sulbactam. Five days after admission the patient now presented with still persisting fever and a non-hemodynamically significant pericardial effusion, which was diagnosed by a transthoracic echocardiogram (TTE). Due to the dynamics and severity of symptoms, a CT scan of the thorax was performed only showing small pneumonic infiltrations in both lower lung lobes. Additionally a blood stream infection with staphylococcus aureus (MSSA) was detected by culture and a transesophageal echocardiography was performed excluding endocarditis. Antimicrobial therapy was rotated to flucloxacillin addressing the bloodstream infection. Further conservative therapy was followed by a slow remission of the pericardial effusion. The patient remained stable regarding his cardiorespiratory function all the time. Three days later an MRI of the heart was performed to exclude myocarditis as a cause for the pericardial effusion. The examination showed pericarditis with pericardial effusion and no sign of myocarditis. Additionally, there were pleural effusions and mildly restricted left-ventricular function.

**Fig. 1:**
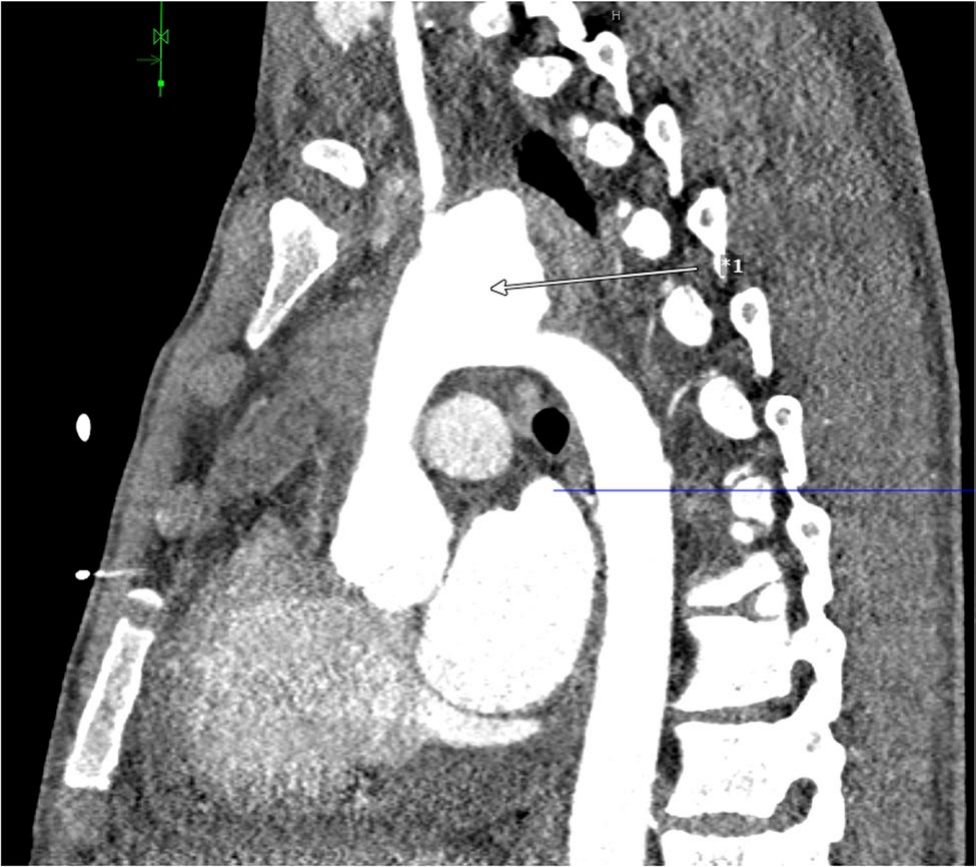
CT scan of the Thorax at the day of operation (sagittal plane), *1 pseudoaneurysm

**Fig. 2:**
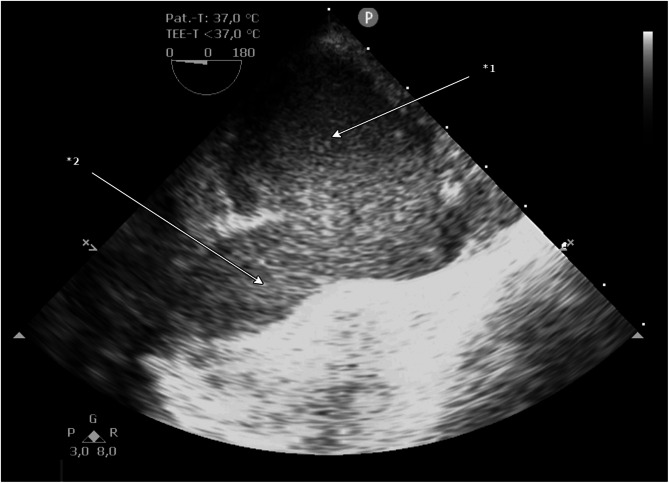
Transesophageal echocardiography of ruptured aorta with the pseudoaneurysm, long axis, *1 pseudoaneurysm; *2 aortic arch

Immediately after the examination, the patient became hemodynamically unstable caused by a very quick progression of the pericardial effusion. Drainage of the effusion by subxiphoid puncture was unsuccessful, so a thoracoscopic pericardial fenestration was conducted via the left chest cavity draining 900 ml of bloody-serous fluid. A routine CT scan the following day showed a 2.5 cm long covered perforation of the aortic arch forming a pseudoaneurysm of 6.5 cm x 4 cm (Figs. 1 and 2). The patient was immediately transferred to our hospital for cardiothoracic surgical intervention. An overview of the timeline of events is summed up below (Fig. 7).

### Diagnostics and therapy

**Fig. 3:**
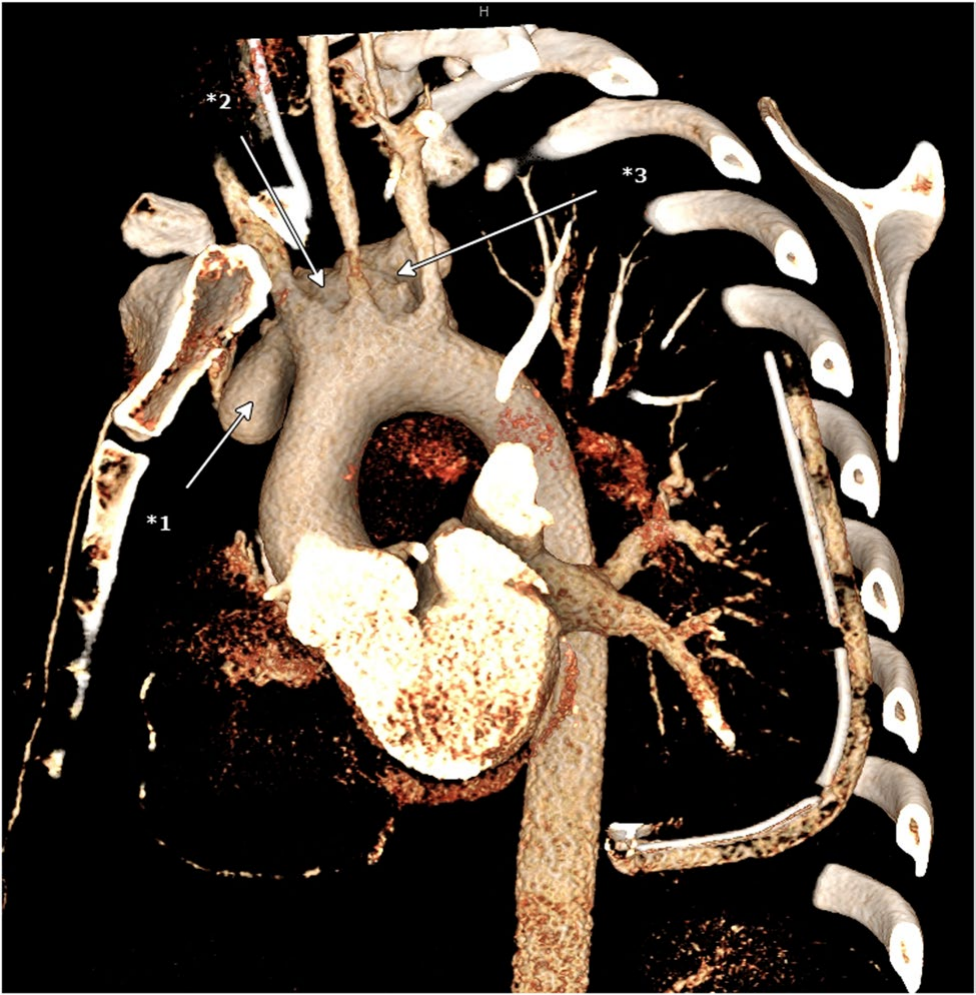
3D reconstruction of the CT scan, *1-*3 pseudoaneurysm

**Fig. 4:**
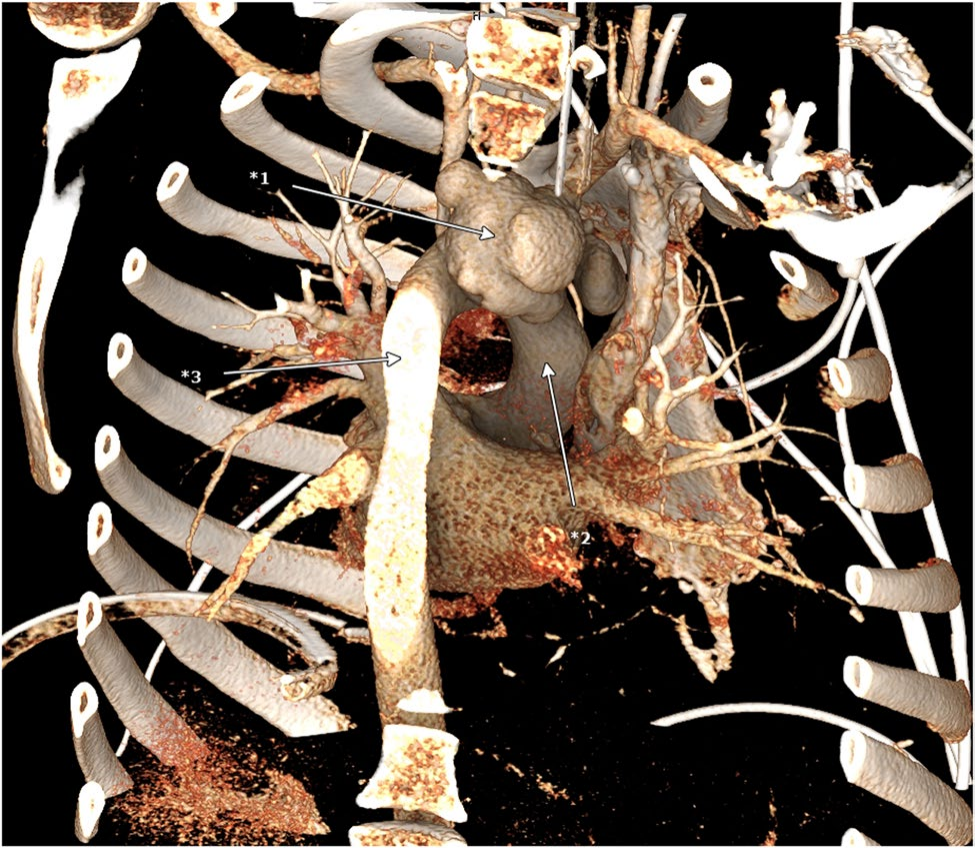
3D reconstruction of the CT scan, view from dorso-lateral, *1 pseudoaneurysm, *2 aortic arch, *3 descending aorta

The patient was admitted to our hospital intubated, mechanically ventilated, and in a stable hemodynamic condition. Blood analysis showed elevated infection markers (C-reactive protein (CRP) 19.1 mg/dl; Procalcitonin (PCT) 8.2ng/ml; white blood cell (WBC) 14 G/l; interleukin-6 (Il-6) 154 pg/ml, fibrinogen 613 mg/dl) as well as markers for an impaired liver function (serum glutamic oxaloacetic transaminase (SGOT) 224 U/l; serum glutamic pyruvic transaminase (SGPT) 212 U/l; International Normalized Ratio (INR) 1.3). The patient was immediately prepared for surgery. The perioperative transesophageal echocardiography (TEE) showed a normal left- and right ventricular function with no valve pathologies (Video 1), the aortic arch showed a rupture over the length of 2–3 cm with a fully perfused pseudoaneurysm of 4 cm × 6.5 cm (Figs. 3 and 4) and (Video 2).

**Fig. 5:**
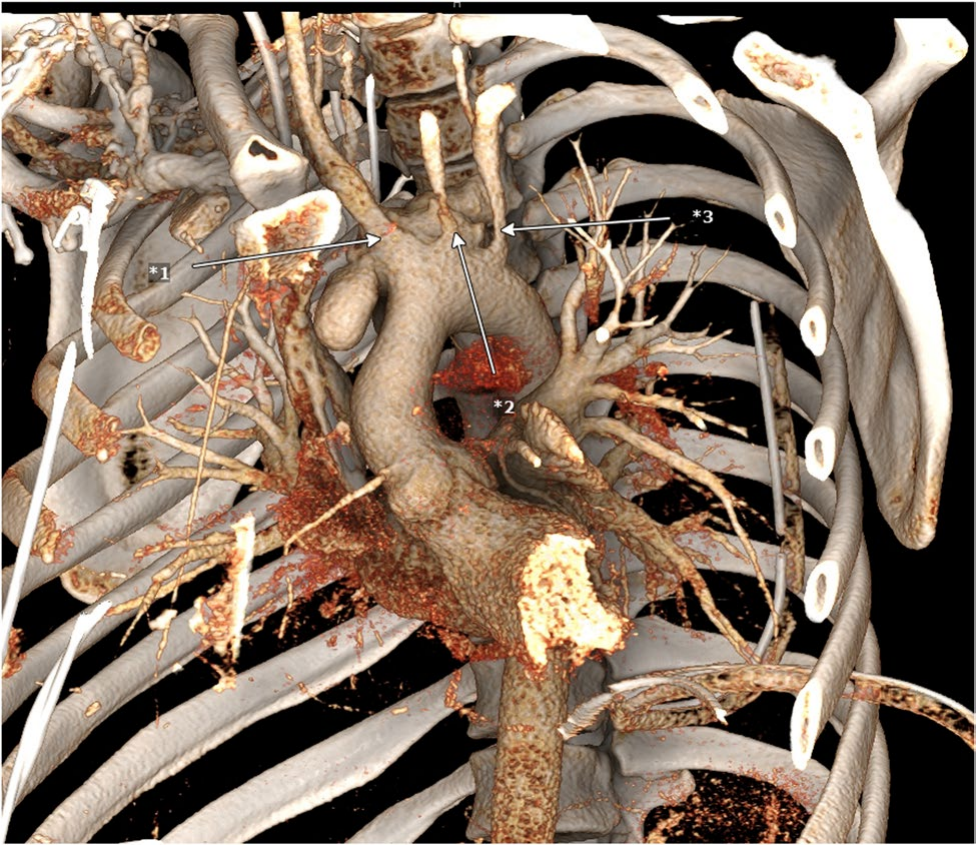
3D reconstruction of the CT scan, *1 brachiocephalic trunk; *2 left common carotid artery, *3 left subclavian artery

**Fig. 6:**
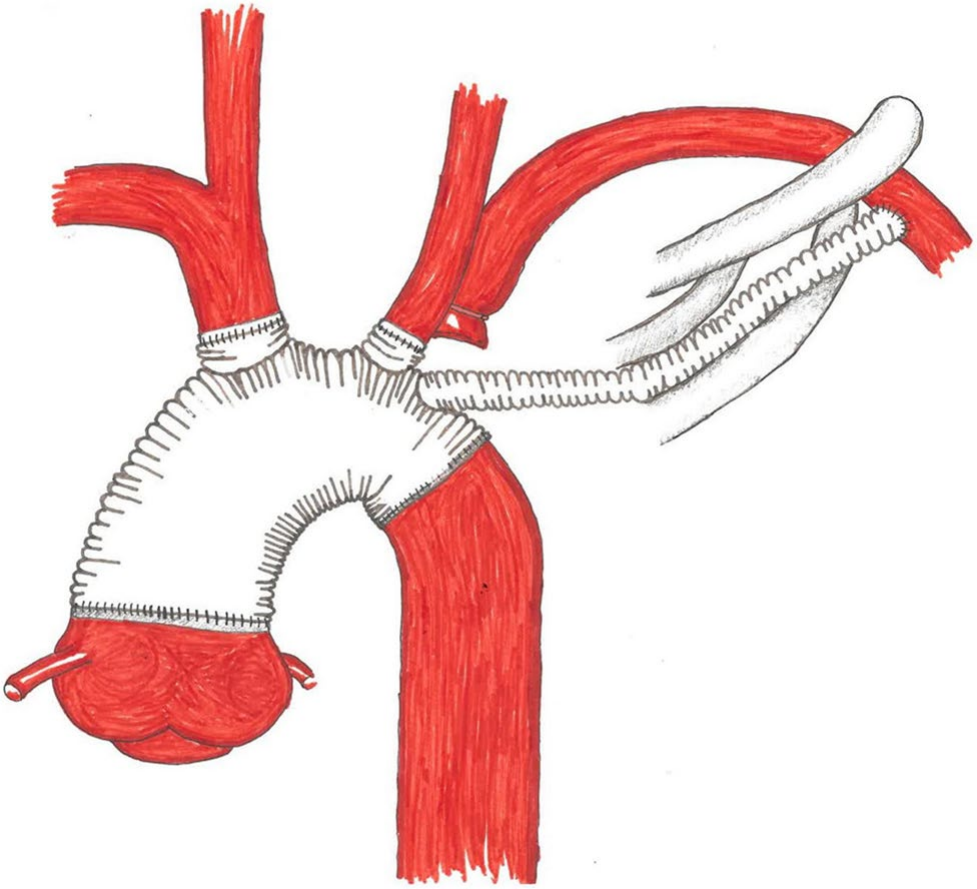
Schematic illustration of the surgical procedure

Under ultrasound guidance, the femoral artery was cannulated using a 17 French cannula, and the femoral vein was cannulated with a 23 French cannula. The patient was cooled down to 24 °C by cardiopulmonary bypass. Then the mediastinum was accessed via a median sternotomy. Noticeably a purulent hemorrhagic pericardial effusion was present. Whilst preparing, the aortic tissue ruptured. The bleed was controlled through manual compression. A hypothermic circulatory arrest was initiated. Selective cardioplegia was administered into the coronary ostia. Upon inspection of the situs the inflammatory rupture had caused tearing of the left carotid artery and brachiocephalic trunk. Perfusion had been upheld through the pseudoaneurysm (Fig. 5). Antegrade brain perfusion was established. The brachiocephalic trunk was excised, and an island was formed out of the left carotid and subclavian artery. After size measuring, a 24 mm branched Dacron Prothesis (Terumo Gelweave Plexus) was selected. The distal anastomosis was fortified through double felt strips. The left carotid artery and the brachiocephalic trunk were anastomosed to the branches of the graft. Given the inflammatory changes of the vessel the left subclavian artery was occluded (Fig. 6). The circulatory arrest was ended after 121 min. The proximal anastomosis was performed by suture at a supracoronary level. After deairing of the prothesis, reperfusion was initiated. Via the left clavicopectoral triangle, the axillary artery was dissected. The third branch of the graft was passed through the second intercostal space and anastomosed to the left axillary artery in end-to-side technique. The patient was successfully weaned off cardiopulmonary bypass. Due to the inflammatory changes of the aorta extensive surgical hemostasis and substitution of recombinant clotting factor concentrates was needed to control the bleeding (Fibrinogen 13 g; Prothrombin complex concentrate 11200 I.E.; Factor XIII 1250 I.E.; Factor VIII 1000 I.E.; eventually Factor VIIa 250.000 IE). In order to compensate for massive blood loss and to maintain adequate perfusion pressure 3250 ml of red blood cell concentrates, 4500 ml of Fresh Frozen Plasma, 1800 ml of thrombocyte concentrate and 2217 ml salvaged and processed blood were transfused. The patient was transferred to the ICU after mediastinal packing and a provisory thoracic closure. The chest closure was performed two days later without further bleeding complications. Microbial diagnostics of intraoperatively resected aortic and pericardial tissue revealed an infection with staphylococcus aureus (MSSA). Histopathological analysis of the tissue showed an ongoing phlegmonous inflammation of the aorta. Antimicrobial therapy was adjusted to cefazolin, fosfomycin and moxifloxacin. All postoperative drawn blood cultures were sterile.

Screening for viral infections, (HBs-antigen; anti-HCV-IgG/IgM; Anti-HIV-1/2 + p24-Antigen, denguevirus-NS1-Antigen; anti-Dengue virus-IgG; anti-Dengue virus-IgM; anti-HAV-IgM; anti HEV-IgG/IgM all negative), rheumatological disease as well as Syphilis remained negative.

The patient was extubated on the second postoperative day, showed no signs of neurological deficit and remained in a cardiorespiratory stable condition. Postoperative blood analysis showed signs of extensive tissue damage (peaks: creatine kinase (CK) 44126 U/l; myoglobin 16978 ng/ml; lactate dehydrogenase (LDH) 2193 U/l) which resolved in the following days. During the first postoperative days thrombosis of the deep veins in the right leg and of veins in the right neck and arm developed. This was most likely an effect of the extensive, intraoperative substitution of coagulation factor concentrates. Therapeutic anticoagulation was initiated. The patient recovered fully and was discharged to a general ward after twelve days.

To exclude possible sites of a reservoir of staphylococcus aureus a combined positron emission tomography (PET) with 217 MBq F-18 FDG and CT scan was conducted 13 days after the operation. No signs of any metabolic active lesion typical for infectious foci or malignancy were detected in neck, thorax, abdomen or pelvis.

The patient was discharged from the hospital 22 days after surgery with an oral antimicrobial therapy of Co-trimoxazole (trimethoprim and sulfamethoxazole) and rifampicin for a recommended duration of six weeks post-surgery. Control PET-CT was suggested after finishing the course of antimicrobial therapy.

## Timeline

(Fig. 7)

**Fig. 7:**
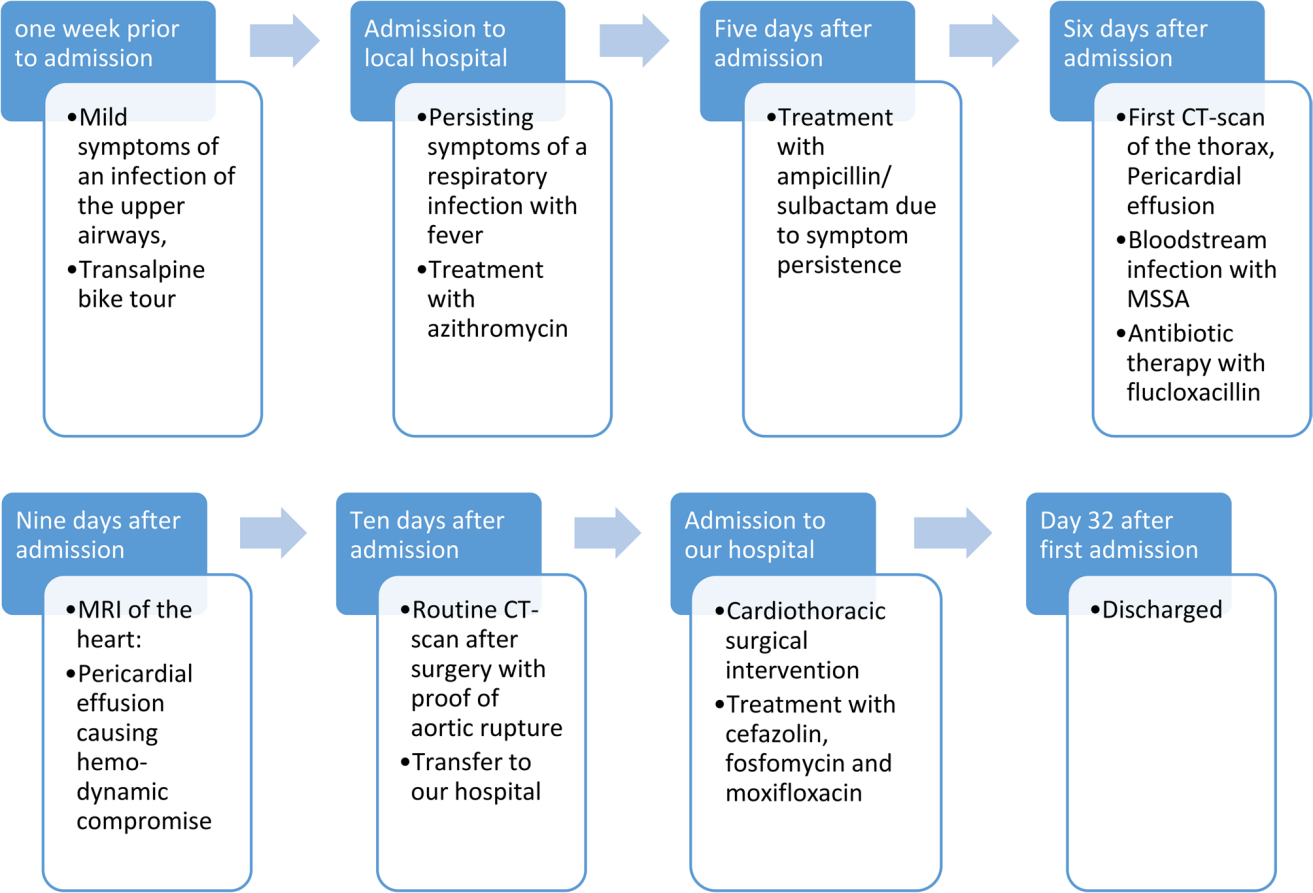
Timeline of patients medical history

### Discussion

Inflammation of the aorta can be classified into two major categories: infectious and non-infectious. While non-infectious aortitis such as primary large vessel vasculitis, Giant Cell Arteritis and Takayasu arteritis are common etiologies, infectious aortitis is a much rarer diagnosis [8]. The aortic intima is generally very resistant to infection, but its disruption can raise the risk of infection [9]. As a consequence, risk factors for the development of infectious aortitis are male gender, age >50 years, atherosclerosis, diabetes mellitus, congenital vascular malformation, history of syphilis and cystic medial necrosis [10]. The most common microorganisms infecting the thoracic aorta are Staphylococcus spp., Enterococcus spp. and Streptococcus pneumoniae [9, 10]. Journeau et al. summed up the most recent case reports with a similar distribution of Staphylococcus aureus, Salmonella spp., Streptococcus spp. and clostridium septicum. They analyzed 55 cases of infectious aortitis and found Streptococcus pneumoniae as predominating pathogen. In addition to the aforementioned risk factors, they observed an association between infectious aortitis and immunosuppression with 22% of their patients with a degree of immunological compromise [3]. 

Most reported cases of infectious aortitis have a common background. The patients affected by this disease are older, suffer from chronic diseases and have some structural vascular anomaly or pathology. This case is distinctive, because the conditions predisposing patients to this disease do not apply to this young, healthy and athletic patient. Thus, we hypothesize that the nearly fatal infection is explainable by the pathogen itself.

In most cases Staphylococcus aureus bacteraemia is caused by the dissemination of pathogens from colonized skin or local skin infections [11]. After entering the bloodstream, the majority of pathogens is eliminated by Kupffer cells originating from the liver and neutrophil granulocytes [12]. In immune-competent hosts, systemic infections are rare, but patients without adequate immune response, especially those suffering from neutrophil dysfunction are very vulnerable to severe and recurrent infections [13]. 

We are proposing two possible explanations for some degree of immuno-suppression. Viral infections of the respiratory tract facilitate bacterial co-infections by various pathogenic mechanisms, such as depressed leukocyte migration, suppressed innate immunity and decreased epithelial barrier function [14]. All of this could have enabled bacteria colonizing mucosa of the respiratory tract to translocate into the bloodstream. Although there is not enough evidence so far to proof a connection between strenuous physical exercise and the susceptibility to respiratory infections, there are several indicators that strenuous physical exercise might increase this susceptibility [15]. To what extent physical strain played a role in the course of the infection in this particular case is merely speculation.

Nevertheless, both viral infection and physical strain might have added up in their immunosuppressive effects and prepared the ground for the further course of the infection. As an evasive mechanism, Staphylococcus aureus is able to migrate into host cells, amongst others endothelial cells, and survive for several days and weeks [16]. Thus, the bacteria might have infiltrated the tissue of the aortic wall by migration. The used β-lactams ampicillin and flucloxacillin have probably no sufficient effectiveness against intracellular staphylococcus aureus [17, 18]. Hence, the infection could spread easily due to an impaired immune function, a pathogen with immune evasive abilities and an insufficient antimicrobial therapy and cause the devastating structural damage to the aortic vessel wall.

## Conclusion

In conclusion, this case shows that even young and healthy individuals can suffer from infectious aortitis without any obvious risk factors. Clinicians should bear that in mind for diagnostic and therapeutic decisions in cases of therapy resistant fever without apparent focus. The point of entry of staphylococcus into the bloodstream might be localized in a disrupted epithelial barrier of the respiratory tract. After entering the bloodstream, staphylococcus aureus was able to spread and to infect the aortic wall. Due to the choice of β-lactams, intracellular bacteria were not effectively controlled and infection of the aorta could progress to the point of rupture. Although the exact pathophysiology remains speculative, we hypothesize the patient had a viral infection of the upper respiratory tract, when he started his bike tour, which, even for well-trained subjects, is an exceptionally intense physical strain. This prepared the ground for an exacerbation of the viral infection and therefore increased the susceptibility for a superinfection by suppression of an adequate immune response.

## Supplementary Information

Supplementary material 1.

Supplementary material 2.

## Data Availability

The raw data generated and analyzed for this report consists of CT-scans, MRI and echocardiographic images. The datasets generated and analyzed during the current study are not publicly available due to size and format of the files, but are available from the corresponding author on reasonable request.

## Abbreviations

MSSA: Methicillin-susceptible staphylococcus aureus
CT: Computed tomography
MRI: Magnetic resonance imaging
TEE: Transesophageal echocardiography
ICU: Intensive care unit
PET: Positron emission tomography
